# Interfacial Interactions and Structural Evolution of Gelatin/Zein Nanofiber Composites Modulated by Poly(Vinyl Alcohol)

**DOI:** 10.3390/foods15132363

**Published:** 2026-07-02

**Authors:** Hui Xiang, Jianhui An, Qin Li, Xinyue Chang, Longchen Shang, Xiujuan Chen, Lingli Deng, Yexing Tao

**Affiliations:** College of Biological and Food Engineering, Hubei Minzu University, Enshi 445000, China; 202430415@hbmzu.edu.cn (H.X.); 2020049@hbmzu.edu.cn (J.A.); karenlovely1026@163.com (Q.L.); changxinyue5757@163.com (X.C.); 2021021@hbmzu.edu.cn (L.S.); xiujuan417@163.com (X.C.); 2019040@hbmzu.edu.cn (L.D.)

**Keywords:** PVA, gelatin, zein, nanofibers, interfacial properties

## Abstract

Synthetic polymers are commonly incorporated into natural polymer nanofibers to enhance their overall performance. In this study, we investigated the effects of different poly(vinyl alcohol) (PVA) concentrations (0%, 2.5%, 5%, 7.5%, and 10% *w*/*v*) on the properties of gelatin/zein nanofibers. With increasing PVA concentration, fiber diameter significantly decreased from 976 ± 165 nm to 262 ± 60 nm, followed by a gradual increase to 396 ± 81 nm, indicating that PVA plays a crucial role in fiber diameter regulation. At higher concentrations (7.5% and 10% *w*/*v*), PVA became dominant, inducing protein aggregation and porous channel formation, which in turn increased the water vapor permeability of the composites. Rheological and mechanical analyses revealed that at these concentrations, the composites exhibited enhanced flexibility while maintaining network stability, demonstrating strong application potential. Furthermore, PVA incorporation induced a slight increase in the primary decomposition temperature (from 320.77 °C to 328.67 °C), indicating enhanced intermolecular compatibility and restricted segmental mobility within the protein–PVA network. Overall, these results establish a theoretical basis for tailoring fiber architecture and interfacial compatibility in natural–synthetic polymer composites. Further, the structural attributes of the resulting fibrous mats indicate their potential for food processing applications beyond conventional food packaging, including use as filtration media.

## 1. Introduction

Ensuring food quality and safety during storage, transport, and distribution relies on packaging, which primarily acts by defending against external agents, prolonging shelf life, and raising product value [1,2]. Recently, electrospun biopolymer-based nanofibers have emerged as highly promising candidates for active and intelligent food packaging materials, owing to their high porosity, large specific surface area, and high loading capacity [3,4,5]. Electrospinning enables precise control over fiber structure and functionality through polymer blending, overcoming the limitations of single-component materials. However, its conventional form suffers from low production efficiency, limiting industrial application [6,7,8,9]. To address this limitation, an air-assisted electrospinning approach has been introduced [10]. By integrating airflow traction, this approach significantly enhances spinning speed and production efficiency, enabling the fabrication of finer, more uniform fibers and offering a viable route to scalable nanofiber production. Cao et al. [11] compared three spinning processes to evaluate their effects on the diameter and jet stability of flame-retardant graphene nanoplatelet composite fibers. They found that air-assisted electrospinning produced highly stable solution jets and yielded fibers with significantly smaller diameters than conventional electrospinning and solution blow spinning.

Among the various raw materials for electrospinning, biobased polymers have garnered significant attention for their biodegradability, renewability, and biocompatibility [12]. Zein, a natural hydrophobic plant protein, exhibits excellent oxygen barrier properties; meanwhile, gelatin offers good biocompatibility and spinnability [13,14]. Blending the two can improve fiber morphology and structural regularity through hydrogen-bonding interactions [15]. In our previous work, gelatin/zein nanofibers loaded with active compounds, such as thymol, eugenol, and dihydromyricetin, were successfully fabricated [16,17]. However, these nanofibers exhibited various limitations, including insufficient mechanical strength, poor stability under humid conditions, and limited functionality, severely compromising their reliability in practical applications such as food packaging. Yang et al. [18] investigated the effects of collagen on the structure, morphology, and spinnability of zein-based nanofibers, finding that its addition significantly improved electrospinning performance. Hesham et al. [19] reported that cospinning gelatin with natural polymers can effectively enhance its mechanical properties, film-forming ability, and stability. Farnaz et al. [20] demonstrated that incorporating nanocellulose during gelatin electrospinning improves mechanical properties and morphological uniformity, providing a promising approach to overcoming its inherent limitations. However, studies on regulating gelatin/zein blend properties using synthetic polymers remain limited.

To address the aforementioned performance limitations, constructing a composite system represents an effective strategy. Poly(vinyl alcohol) (PVA), a water-soluble synthetic polymer, exhibits good biocompatibility and provides the composite material with exceptional mechanical strength and structural integrity through its extensive interchain hydrogen-bonding network [21]. Studies have shown that introducing PVA into other biopolymer matrices can significantly enhance composite performance. For instance, PVA addition can substantially improve the tensile strength (TS) and thermal stability of gelatin/starch systems [22]. In zein-based systems, PVA promotes uniform fiber formation and serves as an effective carrier for active ingredients, such as thymoquinone [23]. He et al. [24] developed PVA/gelatin nanofibers loaded with peppermint oil and amoxicillin, achieving efficient antibacterial activity while reducing antibiotic dosage. However, in-depth studies on how PVA modulates and enhances mechanical properties, structural stability, and active substance loading and release in gelatin/zein binary systems are limited. This knowledge gap hinders the development of high-performance active food packaging materials based on these systems.

Based on these considerations, this study developed a gelatin/zein/PVA ternary system to elucidate the role of PVA in regulating biopolymer structural evolution and interfacial compatibility. We investigated how PVA concentration dictates fiber dimensions and morphological evolution, and their subsequent impact on mechanical robustness and water vapor permeability. Furthermore, water vapor permeability (WVP) measurements were conducted not for direct packaging evaluation, but to probe the intrinsic water transport characteristics of the fibrous network and to establish a quantitative baseline for future barrier-enhancement studies. By analyzing molecular coupling and network stability within the protein–PVA matrix, this study specifically addressed the concentration-dependent mechanism by which PVA regulates molecular entanglement in the gelatin/zein binary matrix. By varying the PVA concentration, we addressed challenges in tailoring fiber architecture and enhancing flexibility, which are often difficult to control in purely proteinaceous systems. Overall, unlike prior studies on macroscopic film properties, this work reveals how PVA-induced protein aggregation and microporous channel formation collectively determine barrier properties and thermal stability in gelatin/zein/PVA ternary nanofibers.

## 2. Materials and Methods

### 2.1. Chemicals

Zein (Z3625) was obtained from Sigma Aldrich (St. Louis, MO, USA), and PVA, gelatin, and acetic acid were supplied by Aladdin (Shanghai, China), a Shanghai-based reagent supplier.

### 2.2. Solution Preparation

**Figure 1:**
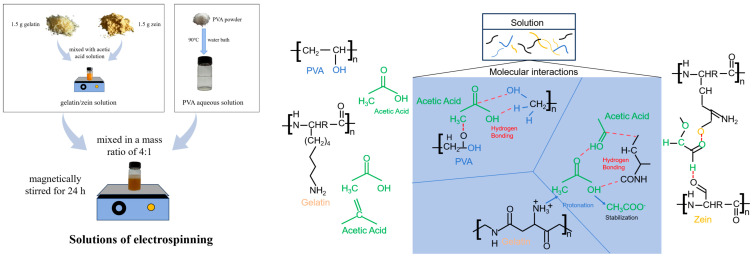
Flow chart of the preparation of PVA/gelatin/zein blended spinning solutions.

The preparation process for the electrospinning solutions is shown in Figure 1. First, a gelatin/zein solution was prepared by dissolving 1.5 g of gelatin and 1.5 g of zein in 87.5% *v*/*v* acetic acid solution (concentration determined based on preliminary experiments). Second, PVA aqueous solutions of varying concentrations (2.5%, 5%, 7.5%, and 10% *w*/*v*) were prepared by dissolving 0.25 g, 0.5 g, 0.75 g, and 1.0 g of PVA, respectively, in 10 mL of deionized water, and then maintained in a 90 °C water bath until complete dissolution. Subsequently, the gelatin/zein solution was mixed with each PVA aqueous solution at a mass ratio of 4:1 and stirred continuously for 24 h using a magnetic stirrer to achieve a homogeneous system. The resulting blended solutions were designated P0, P2.5, P5, P7.5, and P10, where the number indicates the PVA concentration (% *w*/*v*) in the aqueous solution before blending.

### 2.3. Fiber Spinning Process

**Figure 2:**
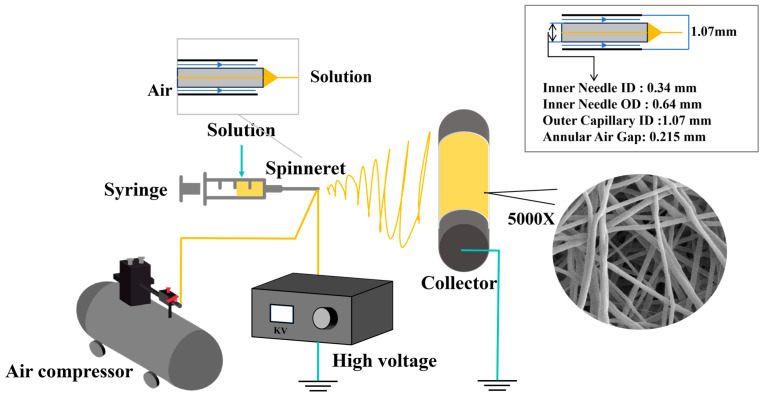
Schematic of electroblowing spinning equipment.

The experimental apparatus for air-assisted electrospinning is illustrated in Figure 2. The system comprised a high-voltage power supply, a programmable syringe pump, a rotating stainless steel grounded collector, and an air compressor. The customized coaxial nozzle consisted of an inner 23G needle (ID: 0.34 mm, OD: 0.64 mm) for polymer solution feeding and an outer 17G capillary (ID: 1.07 mm) for compressed airflow, resulting in an annular air gap of 0.215 mm.

Each solution was processed at a flow rate of 10 mL/h, an applied voltage of 20 kV, and a tip-to-collector distance of 15 cm. An airflow rate of 350 L/h was maintained to provide the necessary aerodynamic shear stress. The methodology was adapted from Chen et al. [25], with minor modifications to the coaxial geometry and gas–electric coupling parameters. Given the small curvature of the needle tip, the local electric field may exceed the dielectric strength of air (nearly 3 kV/mm), potentially inducing localized corona discharge or micro-breakdown. This phenomenon, coupled with the aerodynamic force through the 0.275 mm gap, plays a synergistic role in jet stabilization and solvent evaporation.

### 2.4. Rheological Measurement

Rheological measurements of the spinning solutions were conducted using a rotational rheometer (TA Instruments AR2000ex, New Castle, DE, USA). For each solution, a 1.5 mL sample was loaded onto a 40 mm parallel-plate fixture. The measurements were conducted according to Ji et al. [26], with appropriate modifications.

Steady-state shear test: At a constant temperature of 25 °C, the shear stress (*τ*, Pa) and apparent viscosity (η) of the spinning solution were measured over a shear rate (γ͘) range of 1–200 s^–1^. To quantify the flow characteristics, the *τ*-γ͘ date was fitted to the power-law model:

$$\tau \;=\;K\;\cdot \;{\left(\gamma ͘\right)}^{n}$$

where K (Pa·s^n^) represents the consistency index, and n (dimensionless) is the power-law index.

### 2.5. Fiber Morphologies

Before measurement, a minimal amount of fiber was mounted on conductive adhesive and sputter-coated with gold under vacuum for 45 s at a current of 10 mA. Sample morphology was then characterized by scanning electron microscopy (SEM, Gemini SEM 300, ZEISS, Jena, Germany) at an accelerating voltage of 3 kV and a magnification of 5000×. Fiber diameters were measured from 40 randomly selected fibers per sample using Nano Measurer 1.2 software.

### 2.6. Fourier Transform Infrared (FTIR) Analysis

FTIR spectra were recorded using a Thermo Fisher Scientific Nicolet iS20 spectrometer (Waltham, MA, USA) to investigate chemical and structural changes induced by spinning. Small fragments of the fiber films were compressed into pellets using the KBr pressed-disk method. The method was based on that described by Ali et al. [27], with slight modifications.

### 2.7. Thermal Testing

The thermal characteristics of the fibers were evaluated via thermogravimetric analysis (TGA) and differential scanning calorimetry (DSC). Approximately 5 mg of each sample was heated from 30 °C to 615 °C at a rate of 10 °C/min under a nitrogen atmosphere [28], and the acquired data were processed using Origin 2025 software.

### 2.8. Water Contact Angle (WCA) Measurement

The WCA of the fibers was measured using a contact angle goniometer (Chengde Dingsheng JY-82C Video Contact Angle Measuring Instrument, Chengde, China) to assess the influence of PVA incorporation on surface wetting behavior. Each sample was mounted on a glass slide, a droplet of deionized water was deposited onto the fiber surface, and the process was recorded [29]. Each sample was tested in triplicate.

### 2.9. Surface Elemental Analysis

A sample of appropriate size was mounted onto the sample holder and placed in the introduction chamber of an X-ray photoelectron spectrometer (Thermo Scientific K-Alpha, Waltham, MA, USA). The sample was then transferred to the analysis chamber after the pressure reached below 2.0 × 10^−7^ mbar. Analysis was performed using a spot size of 400 μm, an operating voltage of 12 kV, and a filament current of 6 mA. Survey scans were acquired at a pass energy of 150 eV with a step size of 1.0 eV; meanwhile, high-resolution narrow scans were conducted at a pass energy of 50 eV with a step size of 0.1 eV.

### 2.10. Water Vapor Permeability (WVP) Analysis

The water vapor permeability (WVP) of the fibrous membranes was determined using the gravimetric dish method, adapted from the standard YY/T 0471.2-2004 [30] with adjustment of the test temperature as required, and with reference to the procedure reported by Zou et al. [31]. The membrane thickness was measured at three random positions using a digital thickness gauge and the average value was recorded. A permeation cup containing 10 mL of deionized water was sealed with the fibrous membrane. After recording the initial mass, the cup was placed in a desiccator containing anhydrous silica gel and maintained at 28 °C and approximately 0% relative humidity. The mass was subsequently recorded every hour over a period of 6 h.

### 2.11. Mechanical Properties

The mechanical performance of the fibers was assessed using a DR-508A automated tensile testing machine (Dongri Instrument Ltd., Dongguan, China). Before testing, nanofiber samples were cut into rectangular strips (10 mm × 3 mm) and firmly fixed onto the apparatus. Uniaxial tensile tests were conducted at a loading rate of 5 mm/min with an applied force of 5 N [32]. Five replicates were tested for each sample to ensure result consistency.

### 2.12. Statistical Analysis

Quantitative data, including fiber diameters, mechanical properties, WCA, and WVP, were obtained from at least three independent replicates (expressed as mean ± standard deviation in this paper). Statistical significance was evaluated using one-way analysis of variance followed by Tukey’s post hoc test to determine specific differences between groups. A *p*-value of <0.05 was considered statistically significant. For qualitative characterizations, including SEM morphology, FTIR spectroscopy, TGA, and X-ray photoelectron spectroscopy (XPS), representative results from consistent batches were used to evaluate the structural and thermal properties. All statistical calculations and curve plotting were performed using Origin 2025 (OriginLab, Northampton, MA, USA).

## 3. Results and Discussion

### 3.1. Mechanistic Insights into Air-Assisted Electrospinning

As shown in Figure 2, the needle (outer diameter 0.64 mm) and the grounded outer capillary (inner diameter 1.07 mm) form a coaxial geometry with an annular air gap of 0.215 mm, through which dry sheath air is supplied. Because air is an excellent insulator (relative permittivity ≈ 1, extremely high resistivity), the grounded capillary strongly shields the needle tip. Based on a coaxial cylindrical capacitor model, the actual electric field at the liquid–air interface is therefore significantly lower than that expected at the same applied voltage without the sheath gas [33]. In other words, the apparent 20 kV corresponds to a much milder electrostatic traction on the jet-forming region than the voltage alone would suggest. However, owing to the highly non-uniform field distribution, the maximum electric field at the needle surface reaches approximately 93.02 kV/mm—far beyond the dielectric strength of air (nearly 3 kV/mm). Localized corona discharge is thus very likely to occur near the capillary exit [34]. In gas-assisted electrospinning, such discharge is not necessarily detrimental; instead, it can ionize the gas, generate an ionic wind, and increase the surface charge density on the jet, thereby enhancing stretching and fiber thinning. Moreover, the additional charge injection can help stabilize the Taylor cone and suppress whipping, resulting in improved fiber diameter uniformity [35]. This provides a plausible explanation for why the fiber morphology obtained here is superior to what would be expected solely from the applied voltage. Taken together, the coaxial air gap not only reduces the effective electric stress on the liquid surface through the shielding effect but also likely introduces controlled localized discharge, giving rise to a “weakly discharge-assisted electrospinning” regime. Understanding this mechanism offers valuable guidance for tuning the applied voltage, air gap width, and gas flow rate to achieve the desired fibrous architectures.

### 3.2. Rheological Measurements

**Figure 3:**
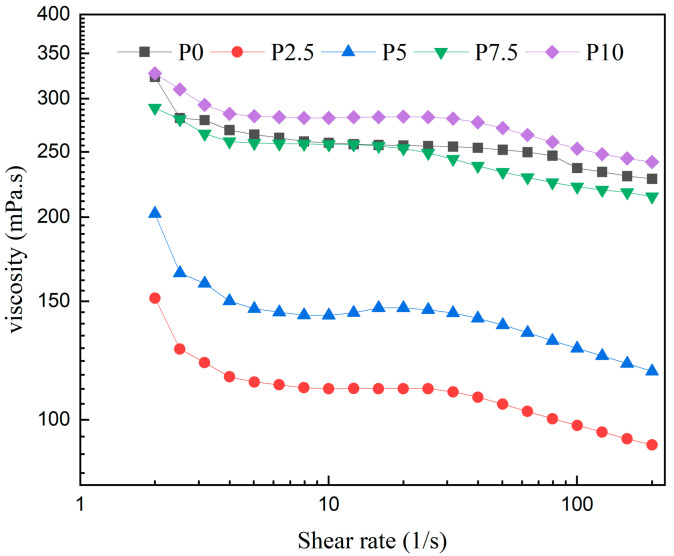
Rheological behavior of gelatin/zein spinning solutions containing different PVA concentrations.

As shown in Figure 3, the apparent viscosities of all samples decreased monotonically with increasing shear rate, exhibiting typical non-Newtonian shear-thinning behavior, which indicates the disruption of internal structures or the orientation of molecular chains along the flow direction under shear. Table 1 summarizes the power-law indices (n) and consistency indices (K) of the composite solutions at different PVA concentrations. The n values of all groups were below 1 (0.88–0.93), further confirming their pseudoplastic fluid characteristics [36]. Among them, the blank group P0 possessed the largest n value (0.93 ± 0.00), which was significantly different from those of the P5 and P7.5 groups (*p* < 0.05), suggesting that the incorporation of PVA slightly enhanced the shear-thinning behavior of the system, although the overall change remained relatively small. Under acidic conditions, gelatin and zein self-assemble into a three-dimensional network via hydrogen bonding and electrostatic interactions, and the viscosity is closely related to the aggregation state of the protein molecules [37]. The consistency coefficient K values of P2.5 and P5 were both significantly lower than that of P0 (0.41 ± 0.09 Pa·s^n^), indicating that low concentrations of PVA can effectively reduce the system consistency and improve its flowability. Notably, the initial decrease in viscosity may originate from the precipitation of small zein particles driven by strong interactions between PVA and gelatin, which would induce phase separation [38]. Since a true solution is expected to be completely transparent, such precipitation would turn the system into a turbid suspension, and a suspension is known to exhibit a lower viscosity than the corresponding homogeneous solution [39]. However, macroscopically, all ternary blend solutions remained highly homogeneous and stable across all PVA concentrations, without any visible turbidity or phase separation, ruling out the possibility of typical protein precipitation or macroscopic phase separation. Therefore, this anomalous initial drop in viscosity is primarily attributed to a “chain conformation contraction and de-entanglement” mechanism, which is driven by the formation of intermolecular hydrogen bonds between the abundant hydroxyl groups (-OH) on the PVA chains and the carboxyl (-COOH), amino (-NH_2_), or -OH groups of gelatin/zein [40]. Specifically, the intense, competitive intermolecular hydrogen bonding between PVA and proteins partially disrupts the pristine protein-protein self-assembly matrix [41,42]. At low PVA loadings (P2.5 and P5), this localized strong interaction forces the extended macromolecular chains to contract into tighter, more compact inter-polymer complex conformations. This dramatic contraction substantially reduces the overall hydrodynamic volume of the biopolymers and lessens the degree of original chain entanglement in the shear field, macroscopically manifesting as a significant decrease in the K value. Moraes et al. [43] also reported that different PVA contents can significantly influence the rheological behavior of gelatin film-forming solutions through hydrogen bonding.

When the PVA concentration was further increased to P7.5, the K value rose to 0.52 ± 0.03 Pa·s^n^, showing no statistically significant difference from P0; the K value of P10 (0.41 ± 0.06 Pa·s^n^) was also comparable to that of P0. This implies that PVA begins to dominate the system at these concentrations: the over-saturated PVA chains overcome the localized chain contraction and initiate extensive bulk-phase intermolecular entanglements throughout the entire ternary matrix. The newly formed hydrogen bonds between PVA, proteins, and water molecules strengthen the physical crosslinking network, slow down chain relaxation, and substantially increase the solution viscosity. It can also be observed from Figure 3 that, in the low-shear-rate region, the apparent viscosities of different samples differed considerably. In particular, P2.5 exhibited a relatively high viscosity, but its viscosity decreased more rapidly with increasing shear rate, reflecting a stronger shear sensitivity; in contrast, in the high-shear-rate region, the curves gradually converged and the differences diminished, which is consistent with the trends revealed by the power-law parameters. These results indicate that low concentrations of PVA can induce macromolecular chain contraction to weaken the interactions within the system and reduce its consistency, whereas higher concentrations of PVA may, through bridging effects or the formation of a more compact three-dimensional entanglement network, conversely enhance the overall strength of the system.

It is worth noting that while the K and n provide critical insights into the steady-state shear rheology and entanglement density of the static spinning dopes, the final morphology of the solidified nanostructures is co-governed by dynamic factors during the flight of the jet.

**Table 1:** Power-law indices (n) and consistency index (K) of gelatin/zein composite solutions incorporated with different contents of PVA.

Group	n	K (Pa·s^n^)
P0	0.93 ± 0.00 ^a^	0.41 ± 0.09 ^a^
P2.5	0.90 ± 0.01 ^ab^	0.15 ± 0.01 ^b^
P5	0.90 ± 0.02 ^b^	0.21 ± 0.05 ^b^
P7.5	0.88 ± 0.00 ^b^	0.52 ± 0.03 ^a^
P10	0.91 ± 0.02 ^ab^	0.41 ± 0.06 ^a^

Note: Different superscripts within the same column indicate values that differ significantly (*p* < 0.05).

### 3.3. Nanofiber Morphology

**Figure 4:**
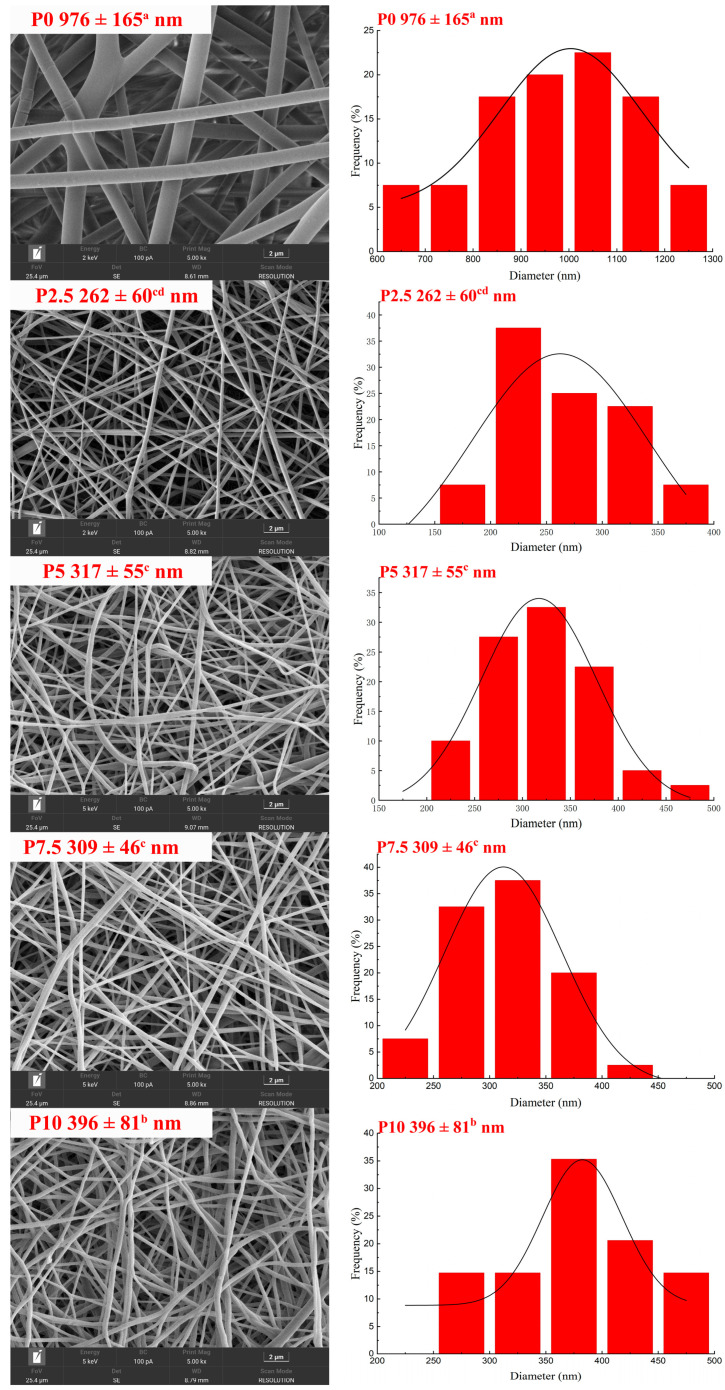
SEM micrographs and fiber diameter distributions of gelatin/zein nanofibers containing different PVA concentrations. Different superscripts within the same column indicate values that differ significantly (*p* < 0.05).

The SEM micrographs of gelatin/zein composite nanofibers with different PVA concentrations and the corresponding diameter distributions are presented in Figure 4. Notably, PVA incorporation induced a drastic reduction in fiber diameter from 976 ± 165 nm to 262 ± 60 nm. This morphological evolution is primarily attributed to the PVA’s disruptive effect on the rigid protein–protein interaction network. Simultaneously, owing to the synergistic stretching effect of the electric field force and auxiliary airflow, the spinning jet experienced reduced resistance to elongation, inducing pronounced tensile deformation and yielding finer fibers [25]. Specifically, PVA chain intercalation weakened the inherent cohesion between gelatin and zein molecules, causing solution viscosity to drop sharply. Chi et al. [44] demonstrated that intermolecular interactions—particularly hydrogen bonding—between PVA and proteins are central to modulating solution rheology and serve as a decisive factor governing fiber morphology and diameter distribution. In this system, the significant viscosity reduction mitigated jet resistance during elongation, thereby facilitating extreme fiber refinement. Conversely, a further increase in PVA concentration caused a diameter rebound to 396 ± 81 nm. Rheological characterization reveals that this recovery was driven by an increase in solution viscosity, which originated from intensified PVA chain entanglement [45]. The reinforced entanglement density enhanced the jet’s resistance to electrostatic drawing, increasing fiber dimensions while optimizing spinnability to yield a highly uniform distribution. Importantly, when correlating these fiber dimensions with the steady-state rheological parameters, the dataset exhibits a distinct mechanistic transition based on the matrix effect of PVA, explaining why a simple monotonic correlation plot is absent. In the absolute absence of the PVA matrix (P0), the spinning dope behaves as a pure protein/bioactive small-molecule variant. Despite sharing an identical steady-state consistency coefficient (K = 0.41) with the P10 group, P0 yields much thicker fibers (976 nm). This decoupling indicates that without the continuous, flexible long-chain entanglements characteristic of the PVA matrix, the P0 jet undergoes completely altered whipping instabilities and rapid solvent evaporation dynamics, solidifying early in a clustered state. Within the PVA-matrix regimes (P2.5 to P10), however, the fiber diameter adheres strictly to a well-behaved, viscosity-dependent increasing trend governed by the concentration-dependent entanglement density.

### 3.4. FTIR Spectra Analysis

**Figure 5:**
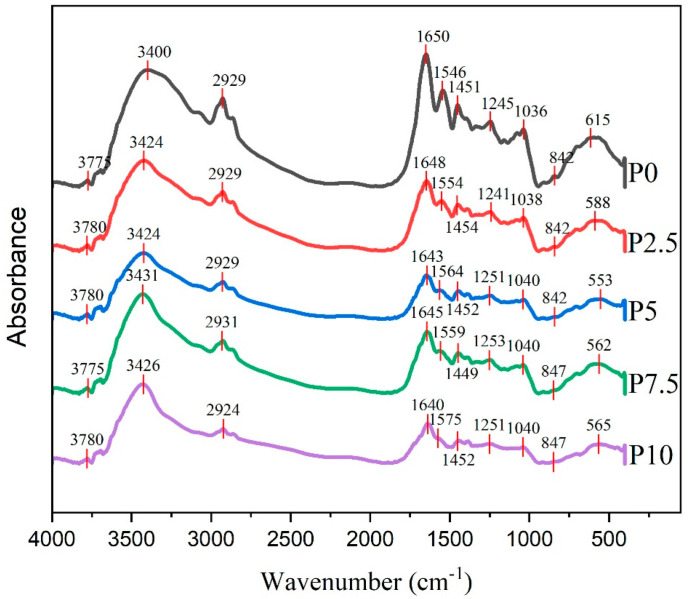
FTIR spectra of gelatin/zein nanofibers containing different PVA concentrations.

As shown in Figure 5, FTIR spectra were used to explore the potential chemical interactions between gelatin/zein and varying PVA concentrations. Characteristic absorption bands were detected at approximately 1650 and 1540 cm^−1^, corresponding to C=O stretching (amide I) and combined C–N stretching/N–H bending (amide II). A distinct band near 1451 cm^−1^ was attributed to the overlap of C–N stretching and N–H bending, while absorption around 1245 cm^−1^ was assigned to the amide III region. In addition, a peak near 1040 cm^−1^ was attributed to C–O stretching vibrations. These results are consistent with those reported by Ullah et al. [46]. In addition, the 1700–1750 cm^−1^ region was specifically examined for evidence of free carboxyl (-COOH) and ester (-COOC) functionalities associated with the solvent matrix and residual acetate units in PVA. As shown in Figure 5, the absence of distinct standalone peaks is likely owing to the rapid evaporation of volatile acetic acid during high-voltage-induced fiber formation. A slight broadening on the higher-wavenumber shoulder of the amide I band (1720–1740 cm^−1^) is observable at higher PVA loadings, hinting at trace-level esterification; however, the overall spectral profile indicates that such chemical grafting is minimal. These results confirm that the structural integrity of the PVA/gelatin/zein nanofibers is primarily governed by a dense intermolecular hydrogen-bond network rather than significant covalent esterification-based crosslinking.

At around 3400 cm^−1^, the broad absorption band originates from the N–H stretching vibration of the amide A band, which typically overlaps with O–H stretching. Notably, with increasing PVA content, this band shifts to 3424 cm^−1^ and ultimately to 3431 cm^−1^. Although hydrogen bonding generally induces a red shift, the observed blue shift in the PVA/gelatin/zein composite indicates a substantial reorganization of the hydrogen-bond network. Specifically, PVA introduction effectively disrupts the original dense intramolecular self-association (strong hydrogen bonding) within the gelatin and zein chains. These original interactions are replaced by extensive intermolecular hydrogen bonds between the −OH groups of PVA and the amide groups (N–H and C=O) of the biopolymers [22]. Although the newly formed intermolecular hydrogen bonds likely possess lower bond energy than the original intramolecular ones, this transformation constructs a more integrated and uniform crosslinked network. Although the newly formed intermolecular hydrogen bonds likely possess lower individual bond energy, their uniform and long-range distribution imposes a more effective kinetic constraint on segmental motion than the original localized intramolecular associations.

### 3.5. Thermal Characteristic

**Figure 6:**
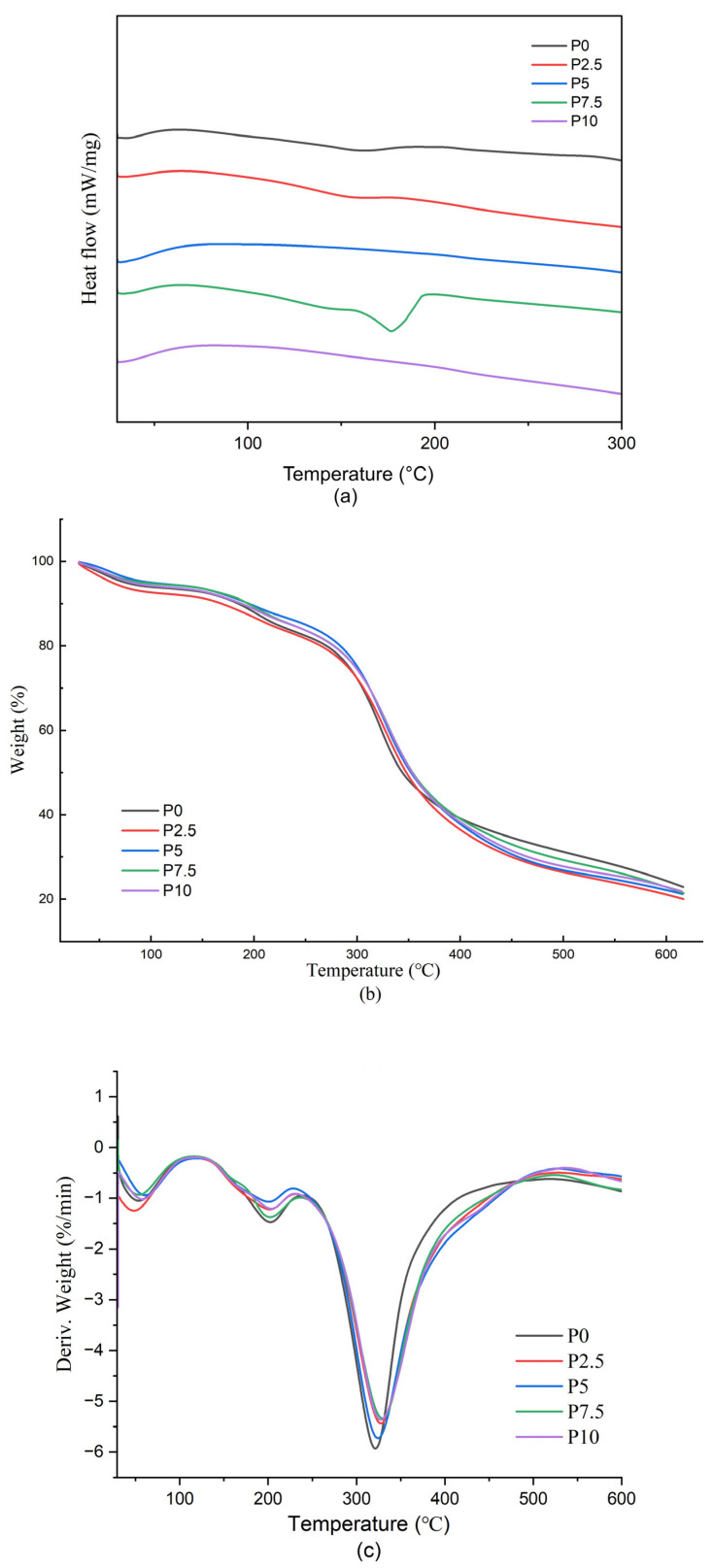
Thermodynamic analysis diagram of gelatin/zein nanofibers at varying PVA concentrations: (**a**) DSC curves; (**b**) TGA curves; (**c**) TGA derivative curves.

Table 2 presents the corresponding TGA data. The effect of PVA content on the thermal stability of gelatin/zein nanofibers was investigated using DSC, TGA and derivative thermogravimetry (DTG) (Figure 6). To further investigate the solid-state thermodynamic behavior and phase compatibility of the blended systems, differential scanning calorimetry (DSC) analysis was performed, and the thermograms are illustrated in Figure 6a. All samples exhibited a broad and mild endothermic peak in the range of 50–120 °C, which was primarily ascribed to the evaporation of bound water within the blend matrix. The glass transition (T_g_) of the polymers might be overlapped and masked by this broad peak, thereby failing to display an independent step-change signal [47]. Crucially, the most prominent feature within the entire tested temperature range was the emergence of a sharp and well-defined crystalline melting peak (T_m_) at approximately 176 °C exclusively for the P7.5 sample. In contrast, the curves of the remaining samples (P0, P2.5, P5, and the higher-loaded P10) remained featureless and flat within this temperature domain, showing no detectable endothermic signals. This sudden divergence in crystallization behavior is in striking agreement with the intermolecular interactions revealed by the solution rheological properties and FTIR spectra discussed in the preceding sections.

For the P7.5 sample, the K reached its maximum value (0.52 ± 0.03 Pa·s^n^), indicating the establishment of moderately ordered, localized self-aggregates within the matrix. This specific architecture not only provided efficient nucleation sites during solvent evaporation and solidification but also did not excessively restrict the segmental mobility. Consequently, the polymer chains were capable of undergoing regular rearrangement to assemble into a relatively perfect crystalline structure, manifested as a distinct melting endotherm on the DSC curve. Conversely, when the concentration was further elevated (P10), this characteristic melting peak completely vanished, and the matrix reverted to a fully amorphous state [48]. At the molecular level, this transition can be elucidated by the robust cross-component intermolecular hydrogen bonding evidenced in the FTIR spectra. In the P10 sample, the amide I band (C=O stretching vibration) shifted to a minimum of 1640 cm^−1^, accompanied by a pronounced decrease in the intensity of the –OH/–NH– vibration bands associated with homotypic molecular association. This indicates that the heterotypic hydrogen bonds were exceptionally intense, which severely suppressed the regular arrangement of homogeneous segments. These robust interactions drove the macromolecular chains into a highly dense and homogeneous amorphous entangled network. Within this rigid network structure, segmental motions were strictly confined, hindering crystallization during the solidification process. This ultimately led to the complete elimination of the melting peak, imparting excellent phase compatibility and amorphous characteristics to the material.

It is worth noting that although previous literature reported the T_g_ of zein to be approximately 140 °C [49], no such transition was detected in the DSC curves of this study. This absence is primarily attributed to a synergistic effect of two factors: first, the weak step-change signal might be concealed by the broad endothermic peak spanning 50–120 °C; second, during electrospinning, the solvent evaporated rapidly within the millisecond-scale whipping jet, which tended to freeze the polymer chains into a highly constrained, non-equilibrium conformation. This constrained state, together with nanoconfinement effects, can broaden the glass transition and shift it away from the expected temperature, making it undetectable in the present DSC scan. Upon the initial heating scan, the relaxation of frozen-in stresses or structural enthalpy frequently causes the T_g_ step to become excessively broadened or even indiscernible [50,51]. Generally, such a non-equilibrium state is unfavorable for the ordered packing and crystallization of molecular chains. Nevertheless, the P7.5 sample manifested a sharp crystalline melting peak, demonstrating that its unique, moderate localized self-aggregation domains afforded the segments sufficient mobility and nucleation capability. This enabled them to partially overcome the non-equilibrium constraints during solidification and develop a well-defined crystalline structure, further confirming the precise regulation of the components’ intermolecular interaction strength on the resulting aggregate morphology.

To further evaluate the thermal stability of the blended systems, the thermal degradation behavior of the samples was monitored by TGA, and the results are summarized in Table 2 and Figure 6b. All samples exhibited three distinct degradation stages. The first stage (below 100 °C) corresponds to the evaporation of adsorbed moisture and residual solvent, which aligns with the broad endothermic peak observed in DSC analysis (50–120 °C) [52]. It should be noted that the mass loss in this stage may include not only water but also trace amounts of residual acetic acid, as a relatively pronounced mass loss was observed around 100 °C in the TGA curves. The second stage (approximately 150–250 °C) involves the initial cleavage of side chains and the decomposition of smaller molecular components within the gelatin/zein/PVA blend. Notably, the weight loss curves below 200 °C remained consistent across all samples, indicating that the trace residual solvent content was comparable among different samples; therefore, interference of residual solvents with subsequent thermal stability evaluations could still be excluded. The third and most significant stage (250–600 °C) represents the primary thermal degradation process, characterized by extensive decomposition of the polymer backbone chains. Notably, as the PVA content increased, the temperature at the maximum degradation rate (Tmax) for this stage shifted upward from 320.77 °C to 328.67 °C. Although this increase is incremental, it suggests enhanced intermolecular compatibility and strengthened hydrogen-bonding interactions between PVA chains and the protein matrix. Such coupling effectively reinforces the structural integrity of the polymer network, thereby improving the thermal resistance of the system [53]; this finding is highly consistent with the hydrogen-bonding signatures identified by FTIR analysis. Furthermore, with increasing PVA content, the char residue at approximately 616 °C gradually rose from 20.02% to 21.88%, indicating that the incorporation of PVA promotes char formation and suppresses complete thermal decomposition.

**Table 2:** TGA data of gelatin/zein nanofibers at varying PVA concentrations.

	TGA						
Group	Peak 1 (°C)	Weight Loss (%)	Peak 2 (°C)	Weight Loss (%)	Peak 3 (°C)	Weight Loss (%)	Residue at 616 °C (%)
P0	53.88	6.01	202.93	10.32	320.77	60.43	22.90
P2.5	48.68	7.36	201.95	8.98	326.92	63.38	20.02
P5	60.38	5.39	201.07	7.73	323.25	65.58	21.22
P7.5	53.47	5.38	202.68	9.56	328.67	63.47	21.42
P10	57.35	6.10	203.67	8.41	326.85	63.49	21.88

### 3.6. Surface Elemental Analysis

**Figure 7:**
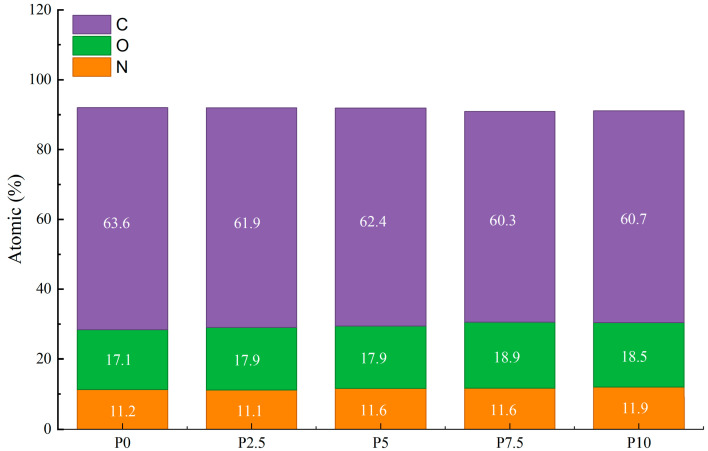
Elemental compositions (atomic%) of gelatin/zein nanofibers containing different PVA concentrations.

**Figure 8:**
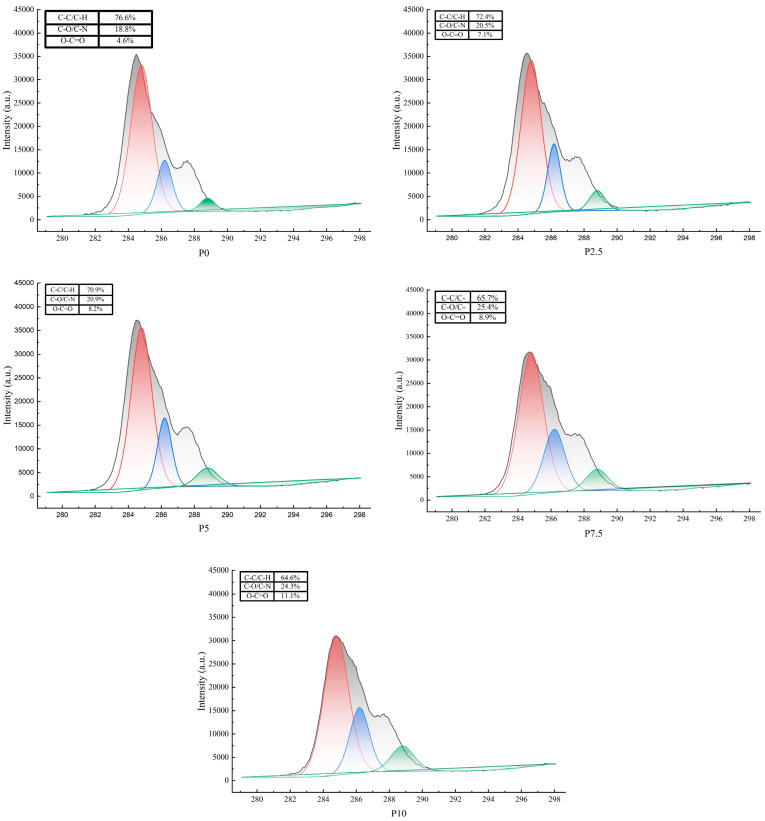
High-resolution C1s XPS spectra of gelatin/zein nanofibers containing different PVA concentrations.

The XPS results are illustrated in Figure 7 and Figure 8. With increasing PVA incorporation, the surface elemental composition of the nanofibers exhibited discernible but relatively restricted variations, with nitrogen content ranging from 11.24% to 11.92%. This is likely attributable to the surface segregation of protein components during electrospinning [54]. Given XPS’s limited probe depth (approximately 3–10 nm), the detected signals predominantly originate from the fiber “skin” rather than the bulk matrix, which, to some extent, masks bulk compositional variations induced by PVA addition [55].

However, the deconvolution of high-resolution C1s spectra (Figure 8) provides semiquantitative, trend-based evidence of the blending effect. With increasing PVA loading, the proportion of C–O/C–N components rose from 18.81% to 24.36%, consistent with hydroxyl group introduction from PVA chains. These findings suggest that although XPS has inherent limitations in the precise quantitative characterization of overall composition in porous fibrous mats, the evolution of functional group ratios qualitatively confirms effective PVA distribution near the fiber surface. Such hydroxyl group enrichment promotes enhanced surface hydrophilicity through extensive hydrogen-bond network formation.

### 3.7. WCA

**Figure 9:**
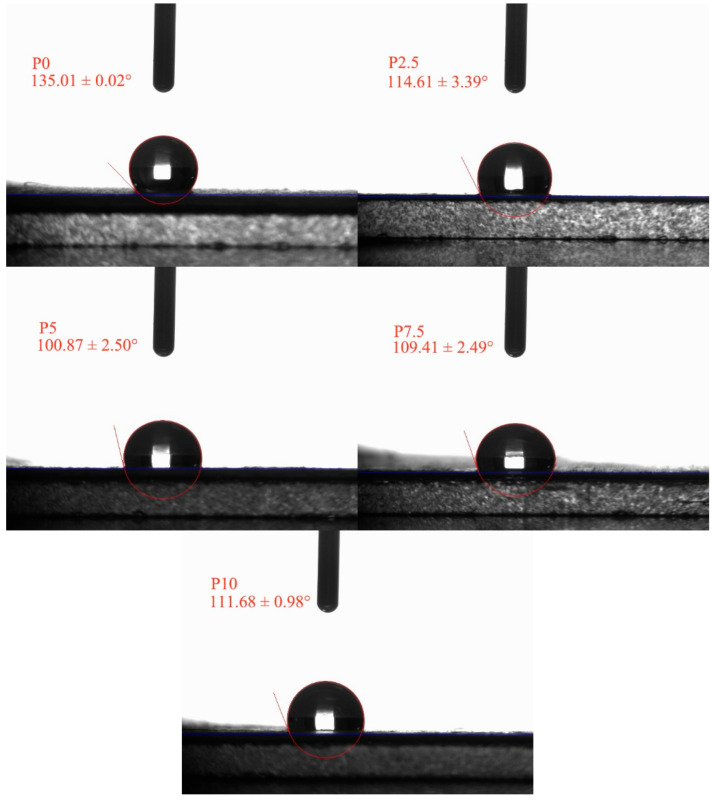
WCA of gelatin/zein nanofibers containing different PVA concentrations.

The WCA measurements of the fibers are presented in Figure 9. A higher WCA corresponds to a more hydrophobic fiber surface [56]. In the pure gelatin/zein system, evaporation of acetic acid during electrospinning alters the solution’s acidic environment, inducing zein self-assembly and microphase-separated protein aggregates within the gelatin matrix [57]. This structure yields pronounced hydrophobicity and a relatively high WCA. Based on PVA’s intrinsic hydrophilicity, its incorporation was initially hypothesized to significantly reduce fiber WCA. Specifically, the WCA decreased to 100.87° with initial PVA addition, which is primarily attributed to the hydrophilic nature of the incorporated PVA on the fiber surface. Upon further increasing the PVA concentration, hydrogen bonding among PVA chains and with water molecules formed a physical network that partially buried surface polar groups, causing a slight WCA rebound to 111.68°. He et al. [58] reported that increasing PVA concentration strengthens the hydrogen-bonding network among polymer chains and with water, thereby reducing surface polar group exposure and modulating the crystallization behavior and surface wettability of PVA/polyphenol composites.

### 3.8. WVP

**Figure 10:**
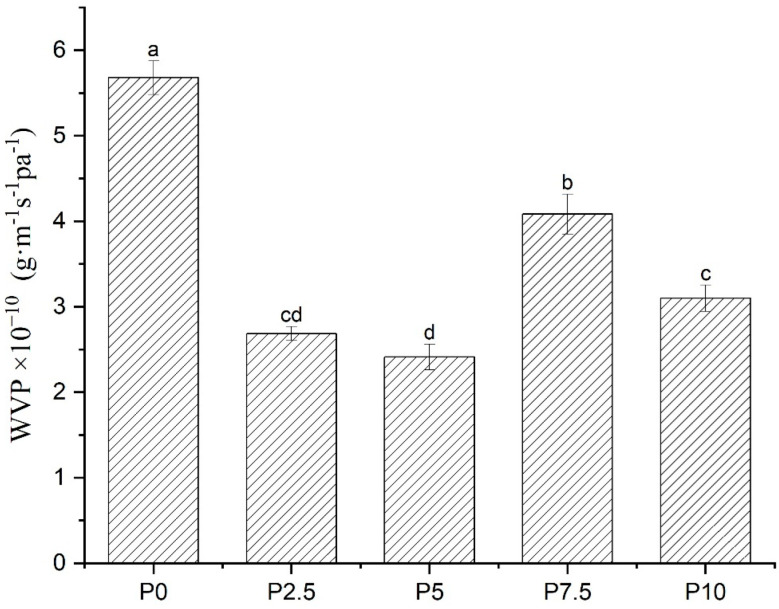
WVP of gelatin/zein nanofibers containing different PVA concentrations. Different superscripts within the same column indicate values that differ significantly (*p* < 0.05).

Table 3 presents the WVP data for PVA/gelatin/zein nanofibers, and Figure 10 shows the dependence of WVP on PVA concentration. It should be emphasized that the WVP measurements in this study were motivated by two fundamental objectives beyond the direct evaluation of packaging barrier performance. First, WVP serves as a sensitive probe of the intrinsic mass transport characteristics of the fibrous network, reflecting inter-fiber connectivity, pore tortuosity, and water–matrix interactions. Such information is essential for elucidating the structural evolution of these hydrophilic fiber systems under high-humidity environments, where moisture-induced swelling and plasticization can substantially alter the transport pathways. Second, although the pristine membranes exhibit WVP values that exceed the typical requirements for high-barrier food packaging, these data establish a systematic baseline against which the efficacy of future barrier-enhancement strategies—such as crosslinking, hydrophobic coating, or composite doping—can be quantitatively benchmarked. Without this baseline WVP dataset, the effectiveness of subsequent modifications cannot be objectively assessed. Collectively, this WVP characterization not only evaluates the immediate barrier performance but also provides a critical foundation for the rational design of structurally optimized, high-barrier nanofibrous materials.

Compared with dense nonfibrous films prepared by conventional casting, nanofiber membranes typically exhibit higher WVP owing to their unique three-dimensional porous structure. Poormohammadian et al. [59] demonstrated that cast films generally possess low porosity and high resistance to water vapor. In the P2.5 and P5 samples, fiber WVP decreased significantly. SEM observations revealed the smallest fiber diameters and a dense network structure at these concentrations. Despite the relatively large specific surface area, reduced interfiber interstices significantly increased the tortuous path for water vapor permeation, making decreased porosity the dominant factor hindering water vapor transport [60]. In the P7.5 and P10 samples, PVA molecules became dominant while interactions with gelatin/zein were insufficient to maintain homogeneous mixing. Consequently, protein aggregation created porous channels, leading to a slight WVP increase, although the overall values remained below those of the pristine fibers.

**Table 3:** WVP data and post-WVP fiber diameter data of gelatin/zein nanofibers at different PVA concentrations.

Group	WVP × 10^−10^ (g·m^−1^·s^−1^·Pa)	Diameter (nm)
P0	5.68 ± 0.196 ^a^	1032.50 ± 151.86 ^a^
P2.5	2.69 ± 0.08 ^cd^	229.75 ± 36.90 ^c^
P5	2.41 ± 0.15 ^d^	221.25 ± 35.46 ^c^
P7.5	4.08 ± 0.23 ^b^	299.25 ± 50.96 ^b^
P10	3.10 ± 0.15 ^c^	284.75 ± 43.26 ^b^

Note: Different superscripts within the same column indicate values that differ significantly (*p* < 0.05).

**Figure 11:**
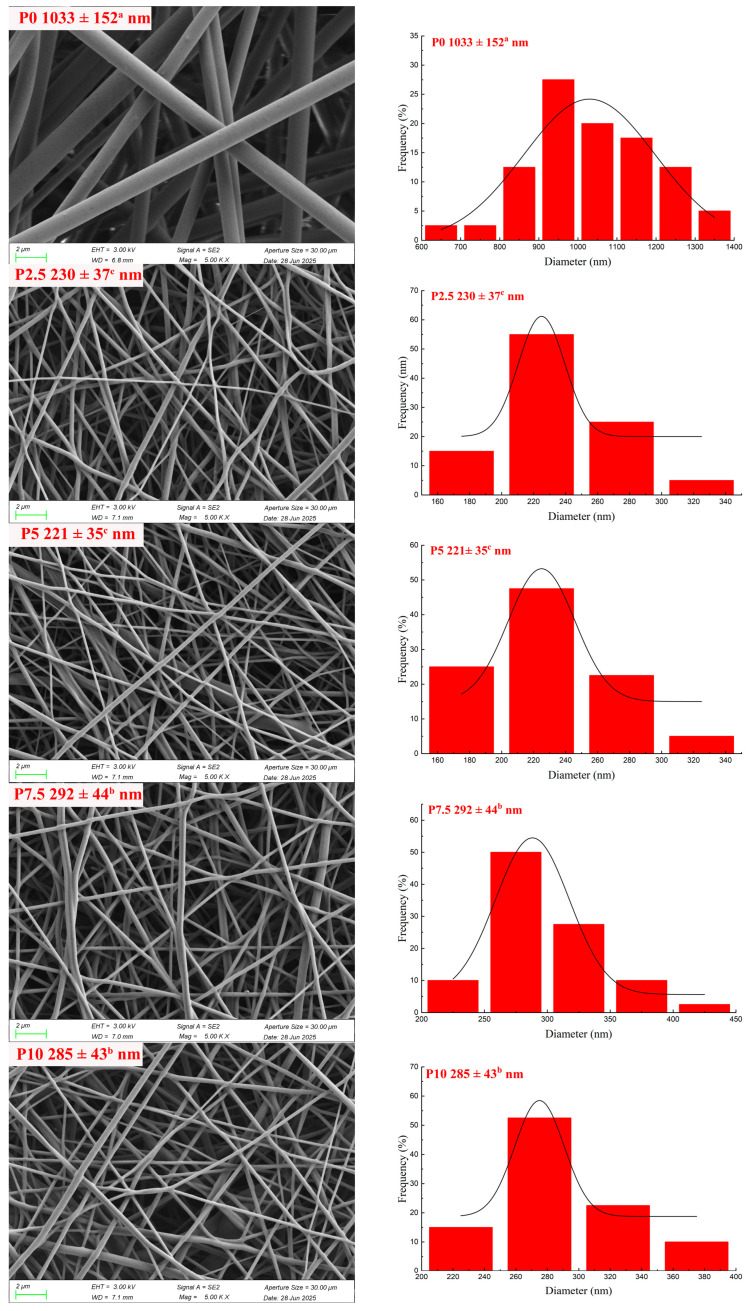
SEM micrographs and corresponding fiber diameter distributions of gelatin/zein composite nanofiber membranes at different PVA concentrations after the water vapor permeability testing. Different superscripts within the same column indicate values that differ significantly (*p* < 0.05).

Table 3 presents the post-WVP fiber diameter data of PVA/gelatin/zein nanofibers. Post-WVP SEM micrographs and corresponding fiber diameter distributions of gelatin/zein composite nanofiber membranes at different PVA concentrations are presented in Figure 11. Following the WVP test, the P0 fiber without PVA exhibited pronounced swelling, which was attributed to its loose amorphous network structure. In stark contrast, all PVA-containing fiber components (P2.5–P10) displayed varying degrees of diameter reduction, i.e., shrinkage. This fundamental behavioral transition reveals the structural reorganization induced by the introduction of PVA: PVA not only acts as a constituent but also constructs a highly crosslinked physical network via intermolecular hydrogen bonds, thereby fundamentally altering the moisture-driven response mode of the fibers. For the low PVA content components, this network already provides sufficient physical constraint to effectively suppress swelling and induce shrinkage.

For the high PVA content components, DSC analysis provided a clear explanation for the structural differences between P7.5 and P10. The DSC curve of the P7.5 sample exhibited a sharp crystalline melting peak at approximately 176 °C, indicating the presence of ordered crystalline domains within the matrix. These crystalline regions served as physical crosslinking points, effectively restricting the movement and expansion of the amorphous chain segments during moisture absorption [61]. In contrast, the P10 sample showed no melting peak or glass transition step across the entire scanned temperature range, suggesting a completely amorphous state. However, a pronounced blue shift in the amide A band (to 3431 cm^−1^) and a distinct red shift in the C=O peak in the amide I region in the FTIR spectrum jointly confirmed the formation of a dense, heterogeneous intermolecular hydrogen bonding network in this sample [62].

Although the aforementioned rigid physical crosslinking networks (whether the crystalline domains in P7.5 or the dense hydrogen bonding network in P10) inherently suppressed water uptake, a small amount of water molecules still penetrated and acted as a plasticizer under the high-humidity conditions of the WVP test [63]. This plasticizing effect facilitated local segmental rearrangement and relaxation of the residual stress induced by rapid spinning. During the subsequent drying process, because the molecular chains had undergone constrained rearrangement under the restriction of the crosslinking network, they eventually packed more tightly, leading to the slight shrinkage of the fiber diameter macroscopically. In summary, these observations demonstrate that the swelling behavior of this fiber system is not solely governed by surface hydrophilicity, but rather constitutes a network-confined relaxation process co-regulated by the hydrogen bonding crosslinking density and the ordered crystalline structure.

### 3.9. Mechanical Characteristics

**Figure 12:**
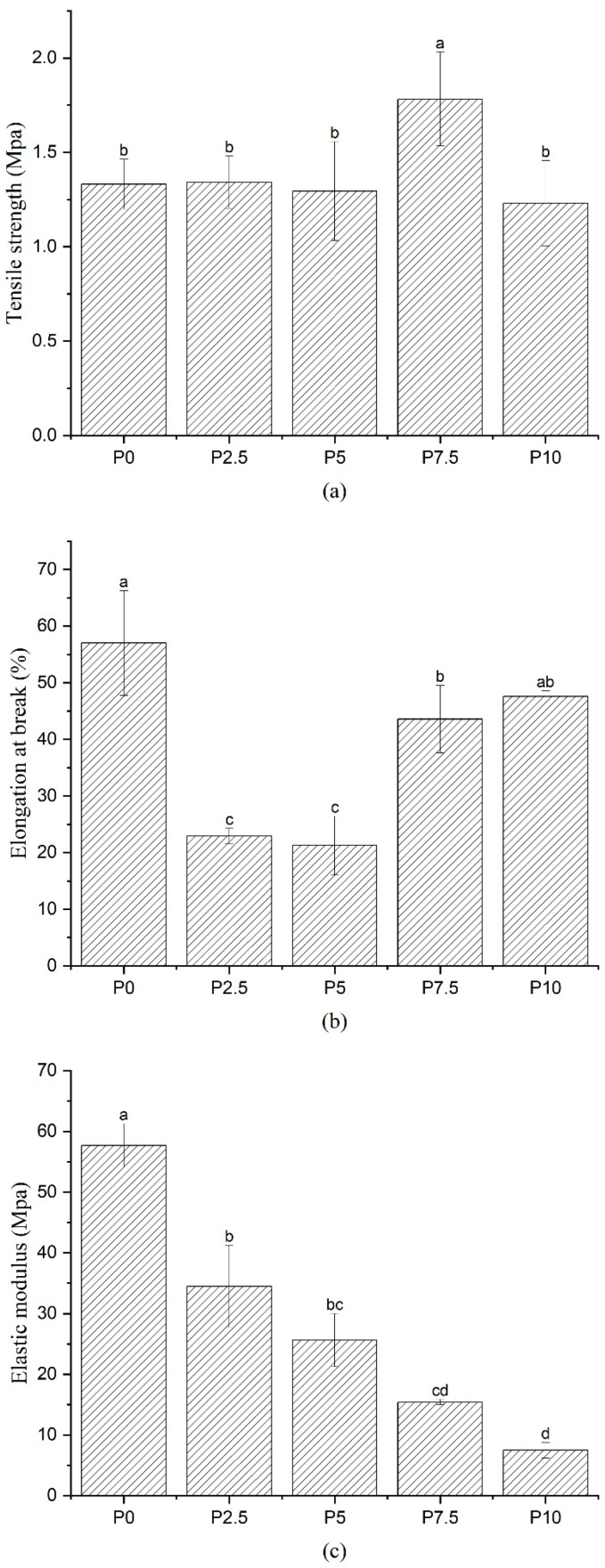
Mechanical properties of gelatin/zein nanofibers at different PVA concentrations: (**a**) TS, (**b**) EB and (**c**) EM. Different superscripts within the same column indicate values that differ significantly (*p* < 0.05).

The effect of PVA addition on the mechanical properties of gelatin/zein nanofibers, including TS, EB, and EM, is illustrated in Table 4 and Figure 12. FTIR analysis indicated weak hydrogen bonding between PVA and the polymer matrix. With increasing PVA content, TS remained largely unchanged or slightly increased, suggesting that mechanical performance is primarily governed by physical chain entanglements rather than chemical crosslinking. At low PVA content (2.5% and 5%), EB decreased, likely because of limited hydrogen bonding restricting chain mobility. As PVA content increased further, weak hydrogen bonds provided some chain mobility, partially restoring elongation [64]. During stretching, these weak hydrogen bonds are readily disrupted, allowing freer molecular chain movement and thereby enhancing fiber flexibility [40]. However, owing to the discontinuous and relatively weak hydrogen-bonding network, EM continued to decline with increasing PVA content. Ishizaka et al. [65] also reported that subtle variations in hydrogen-bond arrangement can significantly influence the mechanical performance of polymeric materials.

## 4. Conclusions

To enhance the applicability of gelatin/zein composite materials in food packaging, varying PVA concentrations were incorporated into the system. Rheological analysis and morphological observation revealed that the appropriate PVA addition increased spinning solution viscosity and molecular chain entanglement, significantly reducing the average fiber diameter from 976 ± 165 to 262 ± 60 nm, effectively eliminating bead defects and improving fiber continuity. FTIR analysis confirmed hydrogen bonding between PVA and the protein matrix. This favorable interfacial compatibility elevated the major thermal decomposition temperature from 320.77 °C to 328.67 °C and significantly improved the mechanical properties of the material, endowing the fiber mat with enhanced flexibility while maintaining network stability. These results indicate that PVA introduction helps achieve a balance between flexibility and strength. Furthermore, although the fiber mats were overall hydrophobic, protein aggregation and porous channel formation at high PVA concentrations (P7.5 and P10) increased WVP. Notably, among all formulations, P7.5 exhibited the most balanced performance in fiber uniformity, thermal stability, and mechanical flexibility, making it the optimal candidate for functional food packaging. However, PVA is not a fully degradable polymer, and its slow degradation rate in natural environments may limit the application of this composite material in the field of fully degradable packaging. Nonetheless, this study still provides experimental evidence and theoretical support for the development of structurally tunable natural–synthetic polymer composite nanofiber mats for food packaging, highlighting the great potential of such materials in degradable functional food packaging.

## Figures and Tables

**Table 4:** Mechanical properties (tensile strength (TS), elongation at break (EB), and elastic modulus (EM)) of gelatin/zein nanofibers at different PVA concentrations.

Group	TS (MPa)	EB (%)	EM (MPa)
P0	1.33 ± 0.13 ^b^	57.02 ± 9.27 ^a^	57.68 ± 3.57 ^a^
P2.5	1.34 ± 0.14 ^b^	22.94 ± 1.32 ^c^	34.50 ± 6.77 ^b^
P5	1.29 ± 0.26 ^b^	21.26 ± 5.19 ^c^	25.64 ± 4.40 ^bc^
P7.5	1.78 ± 0.25 ^a^	43.59 ± 5.98 ^b^	15.40 ± 0.44 ^cd^
P10	1.23 ± 0.23 ^b^	47.58 ± 1.01 ^ab^	7.48 ± 1.28 ^d^

Note: Different superscripts within the same column indicate values that differ significantly (*p* < 0.05).

## Data Availability

The original contributions presented in this study are included in the article. Further inquiries can be directed to the corresponding author.
